# Effect of force direction and impaction angulation during dilaceration impacted central incisor traction: a finite element analysis

**DOI:** 10.1186/s12903-024-04601-2

**Published:** 2024-07-20

**Authors:** Qian Yang, Liu Yang, Ning Li, Kai Sun, Liang Li, Yulan Wang, Xiaohang Han, Tiejun Wang

**Affiliations:** 1 Binzhou Medical University, No. 346 Guanhai Road, Yantai, Shandong 264000 China; 2 Department of Prosthodontics, Binzhou Medical University Affiliated Yantai Stomatological Hospital, No. 142 North Avenue, Yantai, Shandong 264000 China; 3 Department of Orthodontics, Binzhou Medical University Affiliated Yantai Stomatological Hospital, No. 142 North Avenue, Yantai, Shandong 264000 China

**Keywords:** Inverted impacted central incisor, Finite element analysis, Force direction

## Abstract

**Background:**

The effects of traction forces at different angles on impacted central incisors(ICI)with varying inverted angles (IA) may be different. The objective of this study was to analyze the biomechanical effects of different force directions (FD) on developmentally inverted ICI with multi-angle variations and to offer insights and guidance for the treatment of inverted ICI.

**Methods:**

Three-dimensional finite element method was employed to simulate clinical scenarios of inverted ICI traction. As such, 0.2 N of force (direction: antero-superior angles of 90°, 100°, 110°, 120°, and 130° relative to the long axis of the inverted ICI crown) was applied to the inverted ICI with inverse angles (IA) of 40°, 30°, 20°, 10° and 0°. Inverted ICI apical displacement and Von Mises stress on periodontal ligament (PDL) and alveolar bone were compared.

**Results:**

IA and FD showed minimal influence on the stress distribution in the PDL, as higher stresses were concentrated in the apical region. The higher stresses in the alveolar bone are focused on the cervical and apical regions of the tooth. In particular, IA exerts a more significant impact on stress distribution in the alveolar bone than FD. The influence of IA on the apical displacement of inverted ICI is larger than that of FD.

**Conclusions:**

To promote the health of the root and periodontal tissues, it is recommended to use an angle of 100°-110° relative to the long axis of the ICI crown when dealing with a large IA (> 20°) developmentally inverted ICI. Conversely, an angle of 110°-120° can be used.

## Background

Maxillary inverted impacted central incisors (ICI) are a highly specialized subcategory of ICIs [1], with a prevalence ranging from 0.06 to 0.2% [2]. Treating these cases clinically poses significant challenges due to the elevated position of the crowns, resulting in low success rates [2]. The antero-superior angle between the long axis of the crown of an inverted ICI and the plane of the palate referred to as its inverse angles (IA) [3], plays a crucial role in clinical management. As the degree of inversion increases, the complexity of treatment rises and the likelihood of successful outcomes diminishes [3]. The treatment process of ICIs may affect the periodontal tissue, resulting in root and alveolar bone resorption and gingival recession [4]. Furthermore, it can significantly affect the aesthetic appearance and speech of the patient’s anterior teeth [1]. Studies have indicated that genetic, mechanical, and pathological factors contribute to the inverted impacted incisors [5].

Combining surgery with orthodontic traction is a common treatment approach for inverted ICI [6, 7]. However, the treatment of inverted ICIs presents complex challenges. Even after surgical intervention, orthodontic traction may still be required to achieve effective treatment [8]. Prolonged treatment duration, substantial apical displacement, and rotational movements can lead to root resorption and, in severe cases, the extraction of curved inverted ICIs [8, 9]. Lyu et al. [10] and Sun et al. [11] found an improved prognosis with early intervention and lowering of the impacted position for inverted ICIs. Hu et al. [12] found that early intervention can restore the tooth to its normal physiologic position and promote ongoing root development. Hence, achieving precise, safe, and efficient traction for ICIs in early intervention remains a clinical challenge. And, it is crucial to pre-determine the appropriate angle of tractional force to achieve effective orthodontic treatment for impacted teeth.

3-dimensional (3D) finite element modeling and analysis (FEM and FEA) has become an indispensable tool for studying biological systems by examining the stresses and strains applied externally to organisms [13]. Studies suggest that the inverted ICI should be retracted gently and as near to the incisal level of the crown as feasible [14]. However, the optimal force direction (FD) may vary depending on the individual’s IAs. In this study, we aimed to investigate the most effective FD for inverted ICI at various IAs during the developmental period and provide a reference for clinical treatment of inverted ICIs.

## Methods

This study received approval from the Ethics Committee. We obtained cone-beam computed tomography (CBCT) data from a 7-year-old female patient, who had been recommended orthodontic treatment. The CBCT data was collected using a device (version VG; New Tom; Italy). Before this study, we received consent from the patient and her family to use the CT data.

Finite element analysis models: CBCT data were imported into Mimics software (version 21.0; Materialize; Leuven; Belgium) and then extracted preliminary 3D models of the maxilla and a portion of the maxillary dentition and maxilla. The surface models of the periodontal ligament (PDL), cortical bone, and cancellous bone. With smooth and offset functions in 3-Matic software (version 13.0; Materialize), the PDL and cortical bone were modeled with an average thickness of 0.2 mm and 1.5 mm, respectively. An orthodontic accessory was created using SolidWorks (version 2022; Dassault; France).

**Fig. 1:**
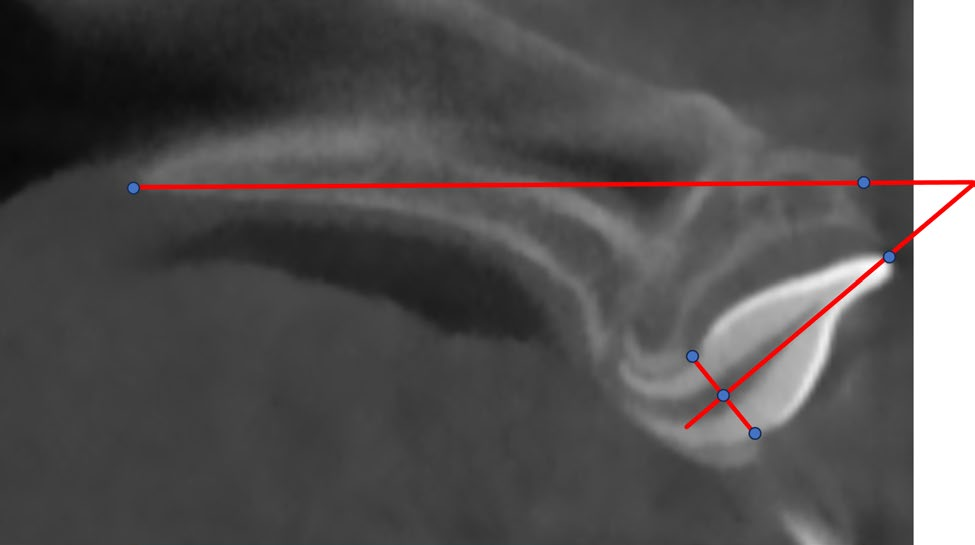
Reference point location and IA in sagittal slices

**Fig. 2:**
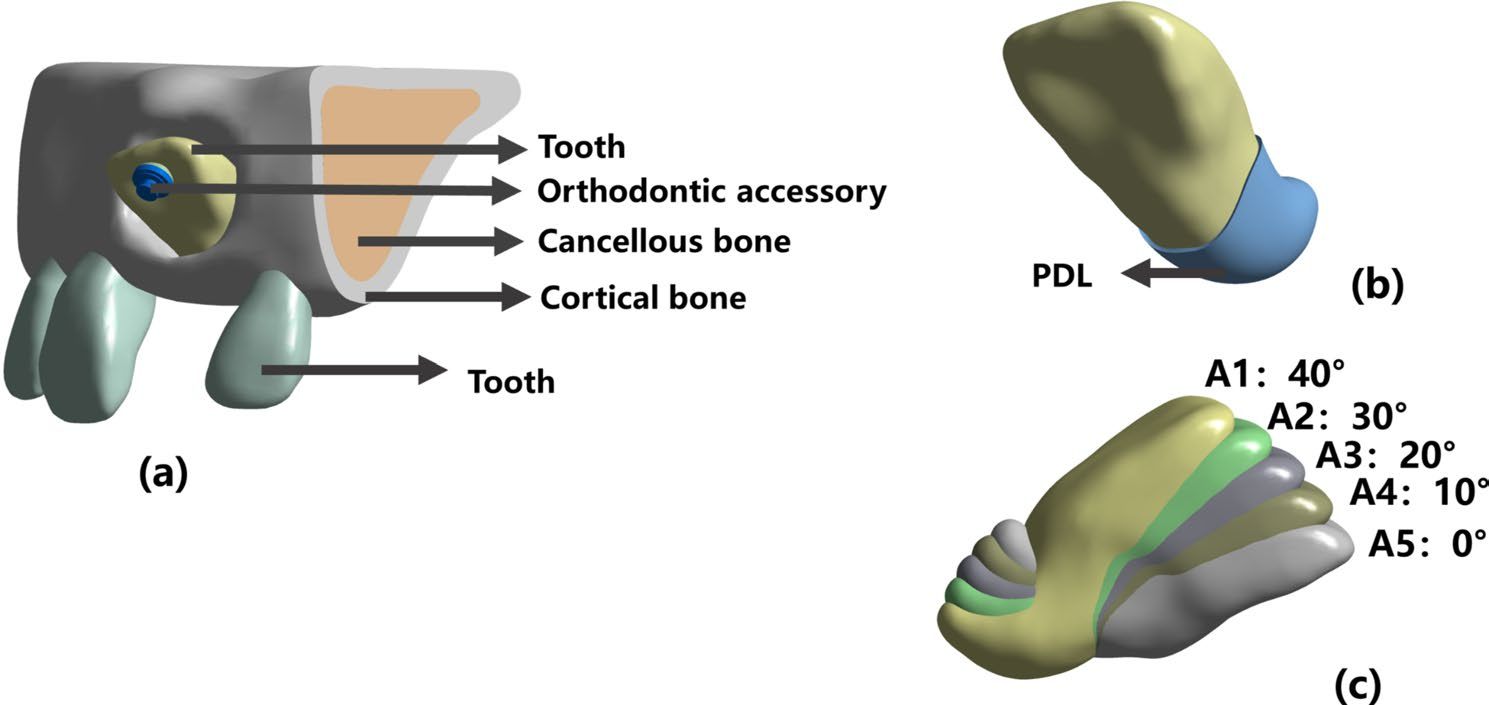
Construction of the finite element model (FEM). (**a**)(**b**) Construction of the FEM of the anterior tooth region containing the tooth, periodontal ligament, and alveolar bone. (**c**) Construction of five inverted ICIs in different positions

The landmarks were established as previously described, and a total of 6 landmarks were selected and are described in Fig. 1; Table 1 [15]. After measurement, the angle of the inverted ICI in this study was 40 degrees. To analyze the best FD for inverted ICI traction with varying IA values precisely, we roughly split IA into five angles (40°, 30°, 20°, 10°, 0°). To obtain five models of different IAs, inverted ICI was relocated in Ansys Space Claim software (version 2021; Ansys; Pennsylvania; USA). Finally, 5 inverted ICI FEMs with 5 distinct IAs (A1, A2, A3, A4, A5) were obtained (Fig. 2). All components, including the maxilla, PDL, teeth, and orthodontic accessory were assembled and imported into Ansys software (version 2021; Ansys; Pennsylvania; USA). Their material properties were assumed to be homogeneous, isotropic linear elastomers [16–18]. Then, the adaptive mesh division method provided by Ansys 2021 software was used to freely divide the mesh. The mesh sizes for tooth, alveolar bone, PDL, and orthodontic accessory were 0.2 mm, 0.3 mm, 0.1 mm, and 0.2 mm, respectively. And, the type of node was tetrahedron. Moreover, material properties and the number of elements and nodes are in Tables 2 and 3, respectively.

**Table 1:** Reference points and variables

Reference point and variable	Definition
UI	Incisal edge of the maxillary central incisor
CEJL	Cementoenamel junction (CEJ) at the labial side
CEJP	CEJ at the palatal side
CEJM	Middle point of the line connecting CEJL and CEJP
ANS	Median, sharp bony process of the maxilla
PNS	Sharp bony process of the hard palate
Inverse angle(IA)	Anterior upper angle between the long axis of the crown and palatal plane, PNS-ANS-UI-CEJM

**Table 2:** Material properties

Material	Young’s modulus (MPa)	Poisson’s ratio
Tooth	1.96 × 10^4^	0.3
PDL	6.9 × 10^− 1^	0.45
Cortical bone	1.37 × 10^4^	0.26
Cancellous bone	1.37 × 10^3^	0.3
Stainless steel	1.14 × 10^5^	0.34
Composite resin	2.2 × 10^4^	0.27

**Table 3:** Nodes and elements

Model	Nodes	Elements
A1	669,082	400,264
A2	669,533	397,736
A3	668,797	398,764
A4	684,865	408,536
A5	675,510	401,814

**Fig. 3:**
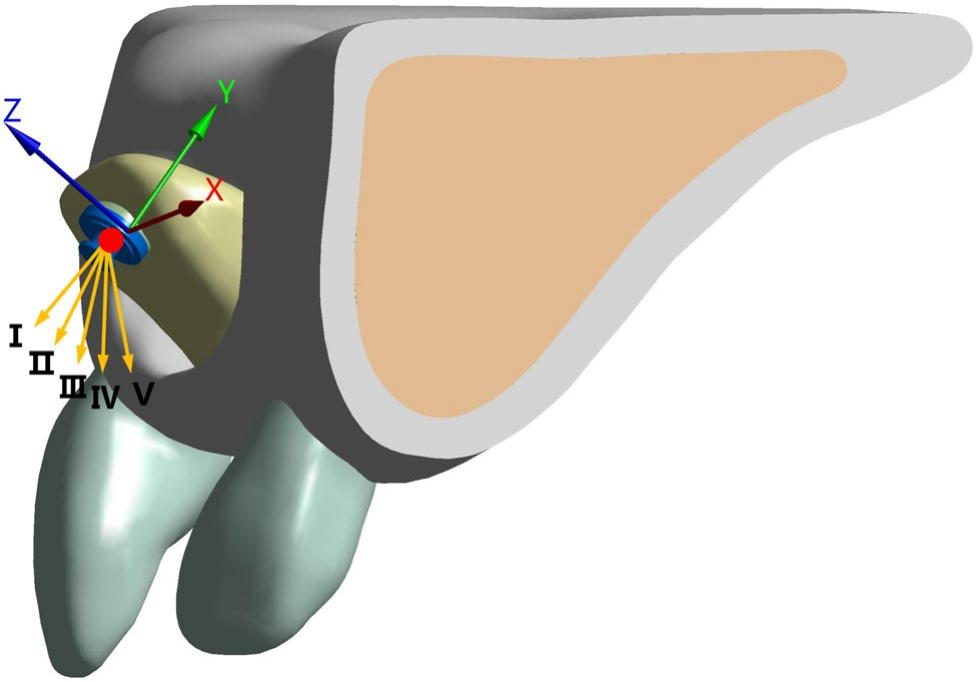
Illustration of the coordinate system and force direction. The establishment method of the coordinate system and the position of the loading force on Five FEMs are the same. The red dot represents the point of the orthodontic force application. The orange arrows represent the direction of the force. Only one model is shown, and the remaining models are the same

Loading and boundary condition: A coordinate system was created to describe the forces exerted on the ICI. The center of the palatal incisal was set as the coordinate origin, and distal direction, buccal direction, and crown direction of the inverted ICI corresponded with the x-, y-, and z-axes (forward direction), respectively (Fig. 3). Considering the final traction position of the ICI, it is unrealistic to expect the anterior-superior angle (between the FD and the long axis of the crown) to be less than 90 degrees. In the clinical environment, due to the controllability of most chain accessories, we only examined the effects of orthodontic attachments [19, 20]. A force of 0.2 N was applied in 5 different FD (I: 90°, II: 100°, III: 110°, IV: 120°, V: 130°): the force simulating the use of chain attachments anchored over the orthodontic appliance (Fig. 3). The left, right, and bottom surfaces of the alveolar bone were set as fixed constraints to simulate attachment to the surrounding maxillary bone. Additionally, bonded constraints were set for teeth and PDL, for PDL and alveolar bone, and orthodontic accessory and inverted ICI.

### Statistical analysis

The Pearson product-moment correlation analysis was used to test the correlations between different IA in Von Mises Stress on PDL, alveolar bone, and apical displacement (y-axis).

## Results

In this study, we compared the stress concentrations in the PDL, the root alveolar bone, and the apical displacement of the ICI in various IAs using different FDs. In particular, we established an independent coordinate system to evaluate the apical displacement to the occlusal plane in the vertical direction, and in the distal and labial-palatal directions, respectively represented by the y-axis, x-axis, and z-axis.

**Fig. 4:**
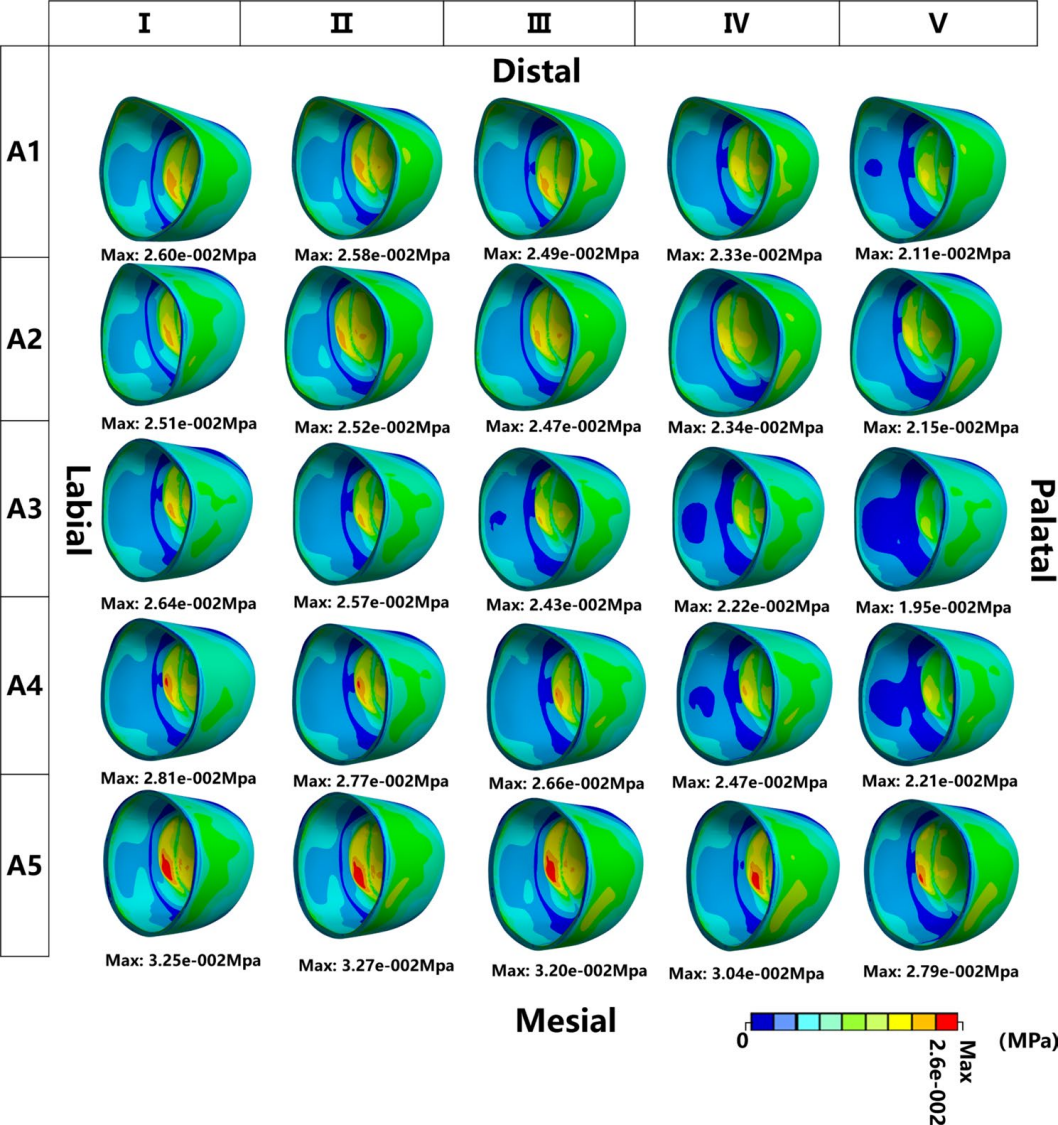
The distribution of the Von Mises Stress on PDL on the FEMs with different IAs and loading conditions. The four direction words in the figure represent the corresponding position of the inverted ICI

**Fig. 5:**
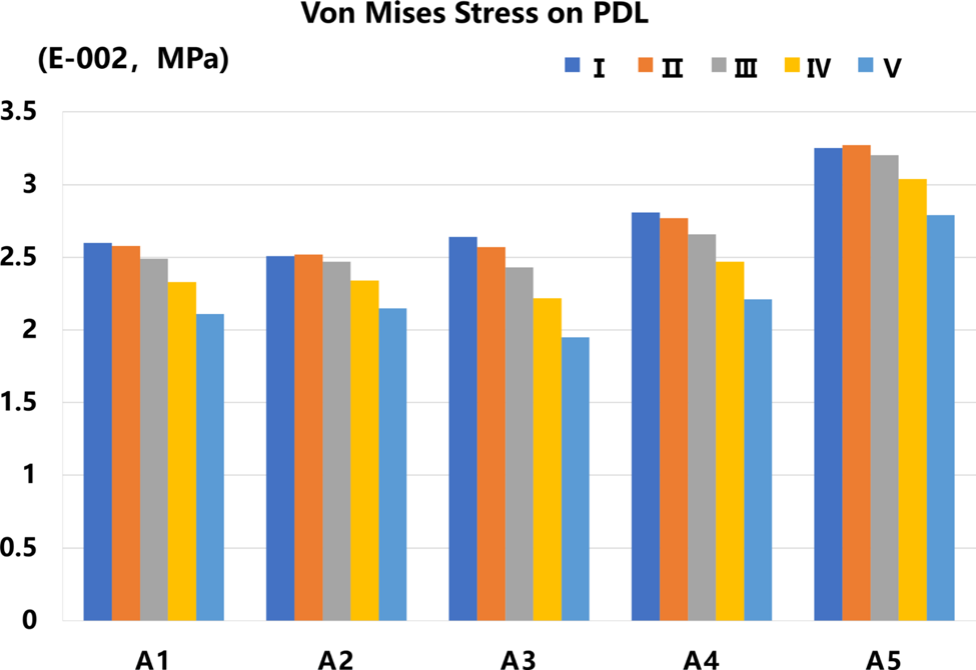
The bar chart of the maximum Von Mises Stress of PDL in different IAs and loading conditions

Von Mises Stress on PDL: In all models, the Von Mises stresses on the PDL were mostly distributed in the root and concentrated in the apical part of the root. Both FD and IA have a relevant effect on the Von Mises stress distribution of the PDL. The r values between any two groups indicated a level of consistency among the results obtained from various IA tests on the PDL stress (*P*<0.05) (Table 4). For a given IA of the inverted ICI, the maximum stress within the PDL showed a gradual decrease or a slight increase as the FD increased. The maximum stress for groups A2 and A5 occurred at a loading force value of II, while for the remaining groups it occurred at a loading force value of I (Figs. 4 and 5).

**Table 4:** The correlation between different IAs in the stress of PDL

	A1, A2	A1, A3	A1, A4	A1, A5	A2, A3	A2, A4	A2, A5	A3, A4	A4, A5
Pearson correlation coefficient, r	0.994	0.996	1.000	0.993	0.980	0.990	1.000	0.998	0.989
*P* value	<0.01**	<0.0001****	<0.0001****	<0.001***	<0.001***	<0.001***	<0.0001****	<0.0001****	<0.01**

*Note* This table reflects the correlation between the maximum stress of PDL with different IAs

*P<0.05, **P<0.01, ***P<0.001, ****P<0.0001

**Fig. 6:**
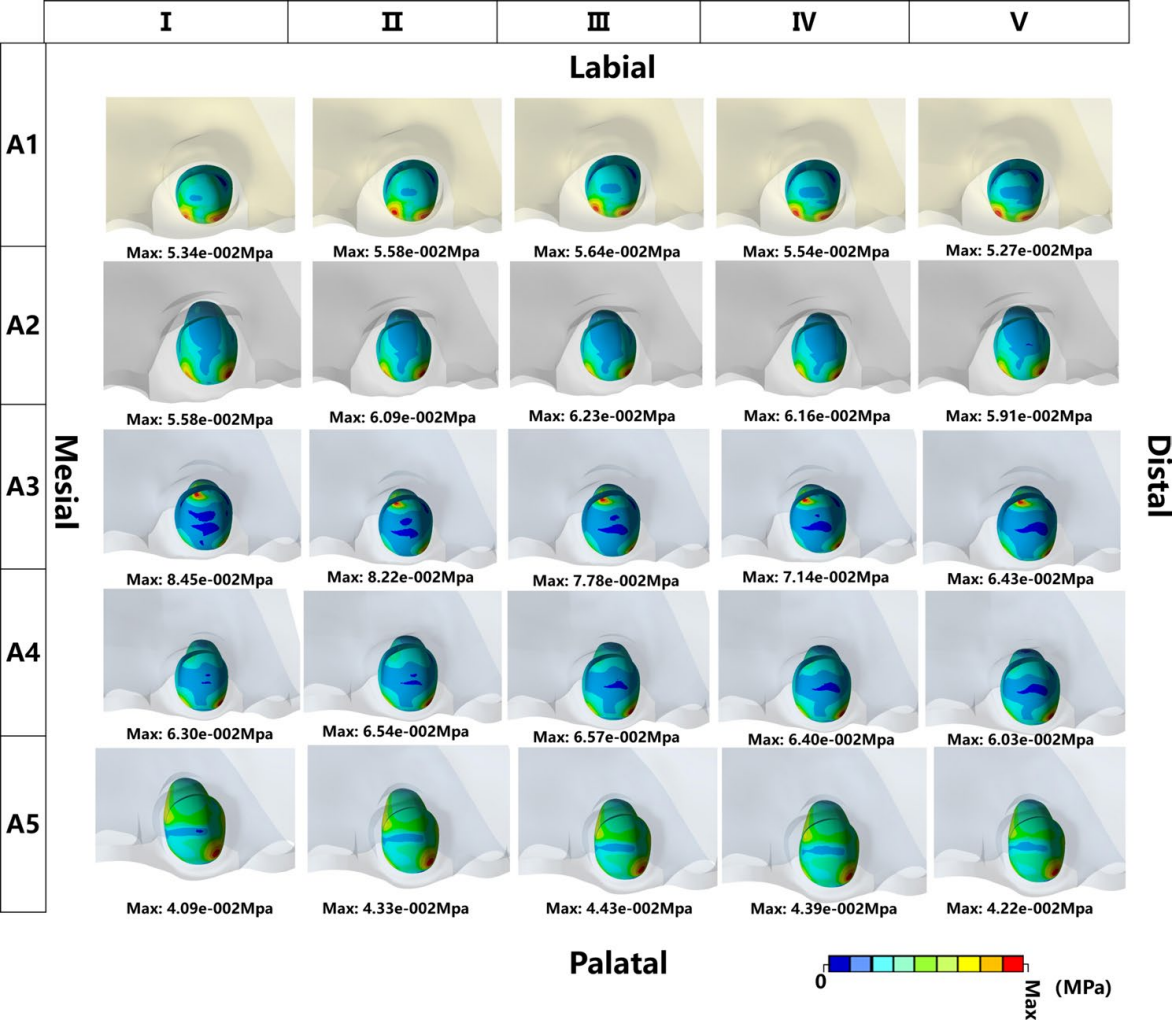
The distribution of the Von Mises Stress on alveolar bone on the FEMs with different IAs and loading conditions. The transparent part is the alveolar bone that is not in contact with the inverted ICI

**Fig. 7:**
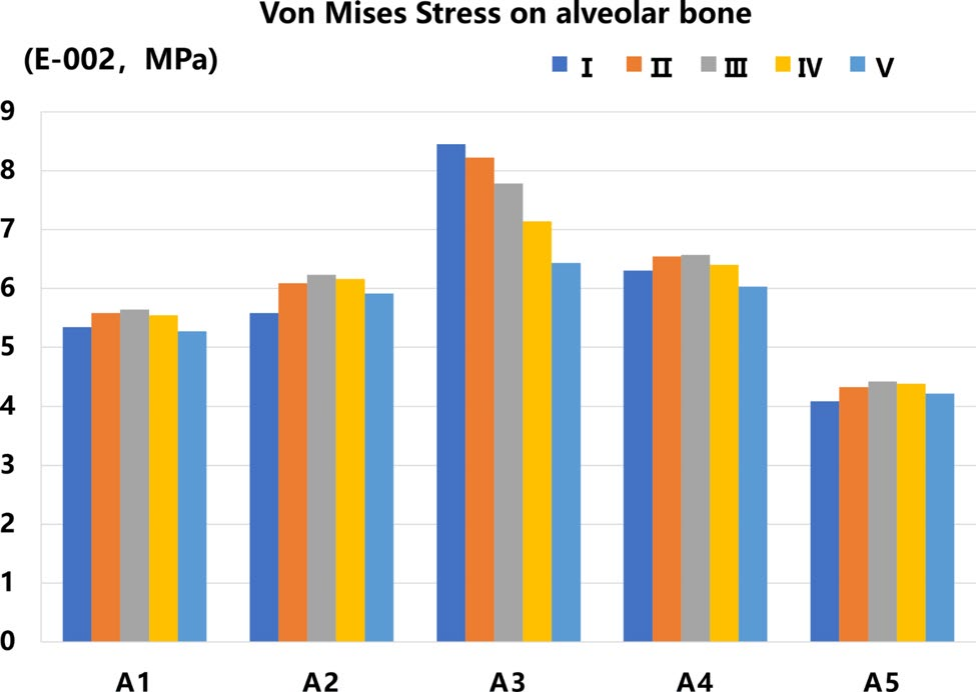
The bar chart of the maximum Von Mises Stress of alveolar bone in different IAs and loading conditions

Von Mises Stress on Alveolar Bone: In all models, the Von Mises stress in the alveolar bone was mostly distributed palatally, concentrated in the cervical and apical regions of the root. FD has less effect on alveolar bone von Mises stress distribution, but IA affects alveolar bone stress distribution. The r value (A1, A4, and A2, A5) suggested a degree of consistency between different IA results regarding the stress on the alveolar bone (*P*<0.05) (Table 5). As IA decreased, the maximum stresses all showed an increasing and then decreasing trend, and all peaked in the A3 group, and the peaks appeared in the apical region of the root. In all models, as the FD increases, there is no increase in the maximum stress. Groups A1, A2, A4, and A5 had the highest alveolar bone stress in Group III. Group A3 has the highest stress in Group I (Figs. 6 and 7).

**Table 5:** The correlation between different IAs in the stress of alveolar bone

	A1, A2	A1, A3	A1, A4	A1, A5	A2, A3	A2, A4	A2, A5	A3, A4	A4, A5
Pearson correlation coefficient, r	0.797	0.360	0.944	0.850	-0.266	0.555	0.990	0.648	0.628
*P* value	>0.05	>0.05	<0.05*	>0.05	>0.05	>0.05	<0.001***	>0.05	>0.05

*Note* This table reflects the correlation between the maximum stress of alveolar bone with different IAs

*P<0.05, **P<0.01, ***P<0.001, ****P<0.0001

**Fig. 8:**
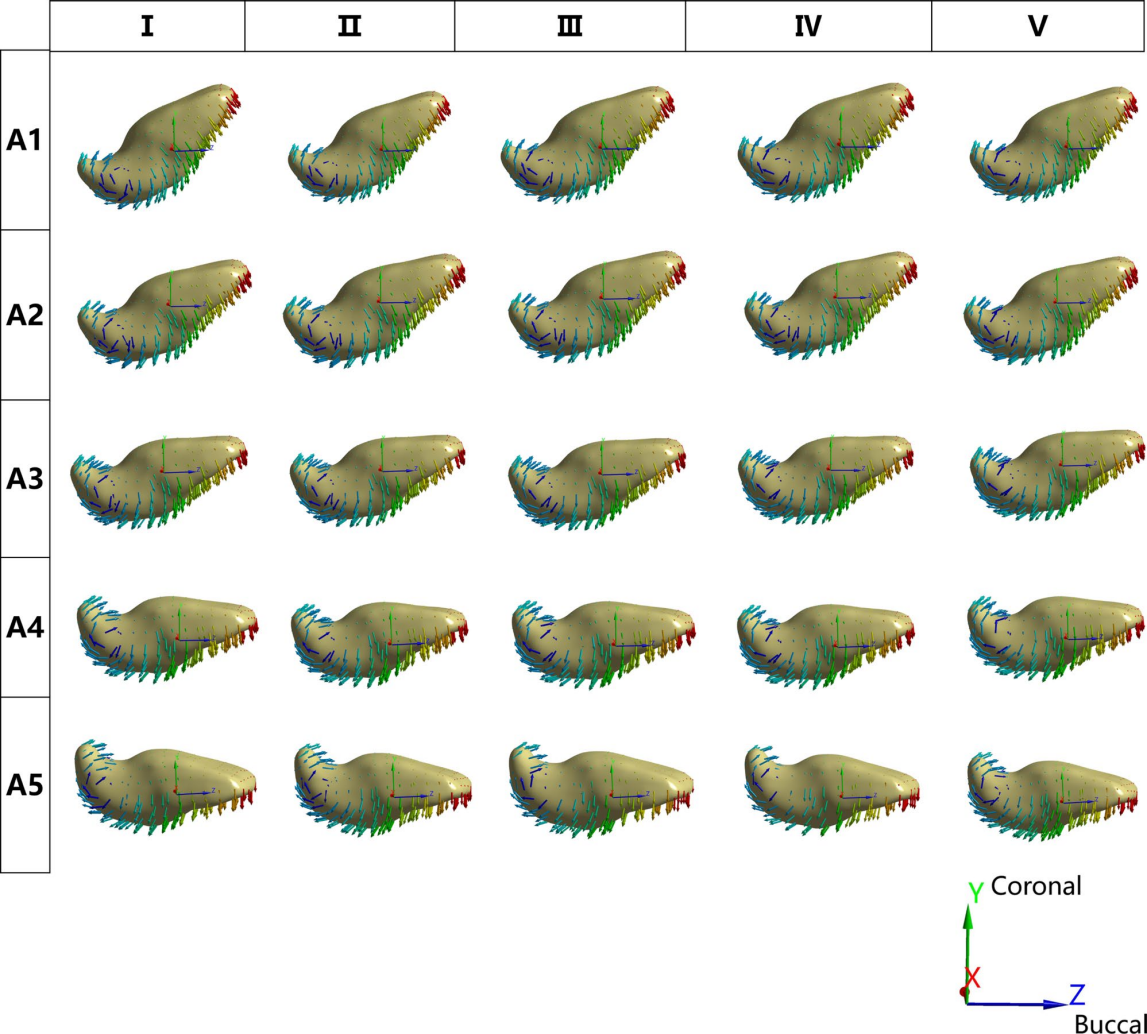
The displacement vector diagram of inverted ICI. The y-axis is perpendicular to the occlusal plane. The movement tendency of inverted ICI

**Fig. 9:**
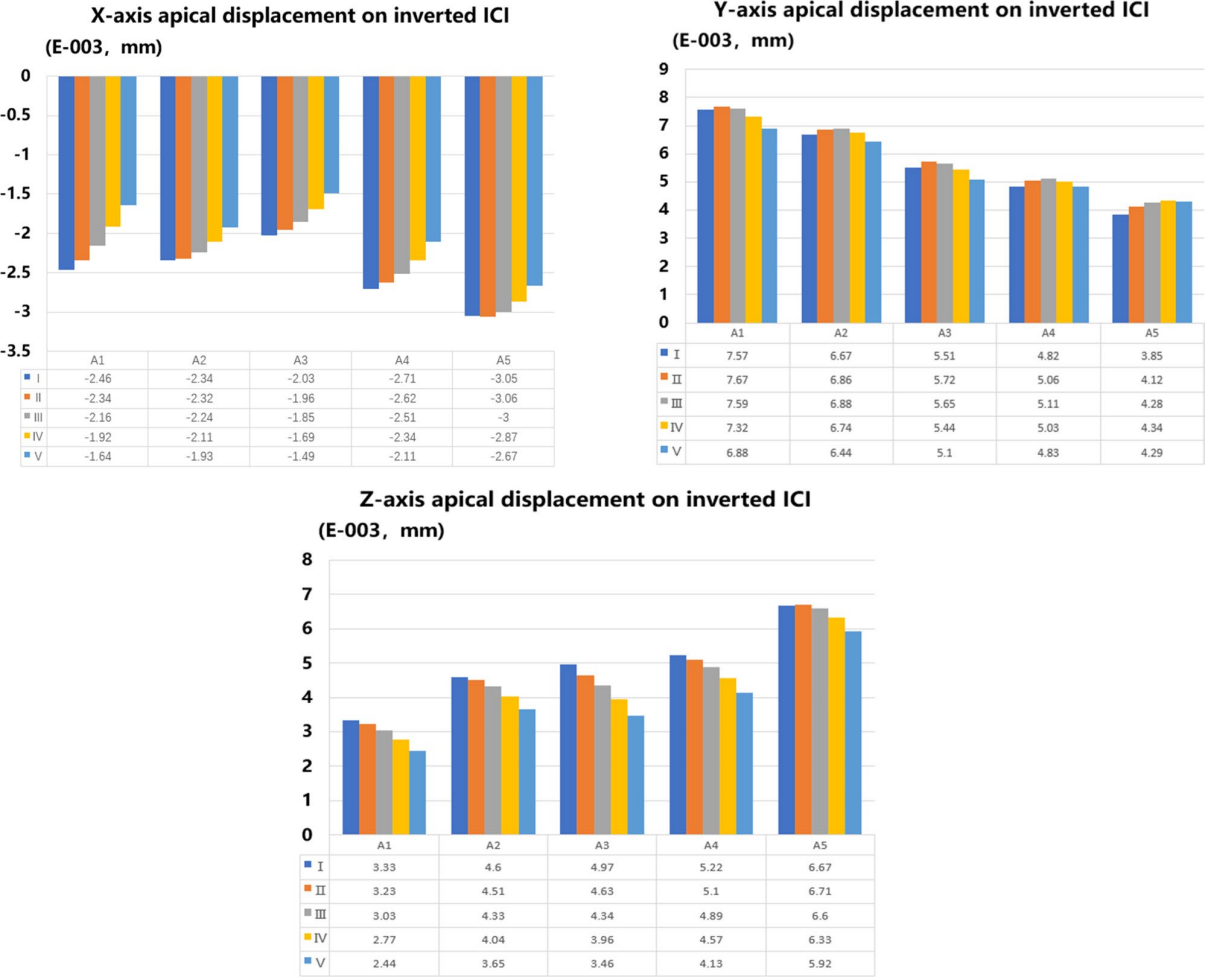
The bar chart of the maximum apical displacement in different IAs and loading conditions. (respectively, x-axis, y-axis, and z-axis)

Displacement on inverted ICI: In all models, FD and IA affect apical displacements. The direction of movement for the inverted ICI was mainly along the y-axis. The r score (A1, A2; A1, A3 and A2, A3) indicated that there was a degree of consistency among the findings from various IA regarding apical displacement (y-axis) (*P*<0.05) (Table 6). The vertical, distal, and labial displacement trends mainly manifested as apical displacement. In the distal direction (x-axis), the A1-A4 groups exhibited a greater tendency to tilt distally at I. In the A5 group, as the FD decreased, the root tip showed a reduced tendency to tilt distally. For the y-axis (vertical to the occlusal plane), as the angle of the traction force increased, the apical displacement of the inverted ICI with different IAs showed a trend of increasing first and then decreasing, except for A5. The maximum apical displacement of the A1 and A3 groups was observed when FD was 100° (II), while for the A2, A4, and A5 groups, it was observed when the traction direction was 110° (III). For the z-axis, as FD increases, the degree of apical inclination towards the labial decreases (Figs. 8 and 9).

**Table 6:** The correlation between different IAs in the apical displacement

	A1, A2	A1, A3	A1, A4	A1, A5	A2, A3	A2, A4	A2, A5	A3, A4	A4, A5
Pearson correlation coefficient, r	0.890	0.974	0.535	-0.478	0.960	0.860	-0.025	0.701	0.482
*P* value	<0.05*	<0.01**	>0.05	>0.05	<0.01**	>0.05	>0.05	>0.05	>0.05

*Note* This table reflects the correlation between the maximum apical displacement (vertical to occlusal plane) with different IAs

*P<0.05, **P<0.01, ***P<0.001, ****P<0.0001

## Discussion

It is well known that the treatment of inverted ICI is a difficult challenge in clinical orthodontics. The labial impacted central incisors are divided into two categories: normal (horizontal impaction, vertical impaction), or inverted vertical position [21, 22]. The treatment of ICIs in the normal position is less challenging. Inverted ICIs are often accompanied by dilaceration, and the majority of the dilaceration angles are obtuse angles [23]. As the crown-root angle decreases (below 90 degrees), the prognosis becomes increasingly unfavorable, and the therapeutic significance is diminished [7, 15]. Orthodontic forces cause stress concentrations on the root surface, which can lead to microcracks on the root surface and root resorption [24, 25], especially for ICI [26]. Previous studies have shown that because the roots of inverted ICI teeth are mostly underdeveloped and accompanied by dilaceration, the traction force should not exceed 0.3 N [2, 6, 26]. Therefore, this study selected a 7-year-old patient with ICI in the middle stage of development and obtuse dilaceration angle.

Insufficient force applied to the PDL will not elicit periodontal tissue reaction or the reaction efficiency will be too minimal. Conversely, excessive force can cause harm to the PDL, loading to root resorption. In most studies, the periodontal ligament is presumed to be a homogeneous, isotropic, linear elastic body [16–18]. In reality, tooth tissue is a homogeneous, isotropic linear elastic body. A multitude of studies [27, 28] have indicated that for loads below 1 N, due to minimal deformation and displacement, all tissues exhibit linear elasticity and isotropy. Consequently, this study also considers the material properties to be homogeneous, isotropic linear elastomers. Based on the experimental results of Wu et al. [28], it can be inferred that the optimal stress for the PDL should be less than 51.2 KPa. The research results of this study are consistent with it. However, there is still a lack of information regarding the most suitable range of PDL when a small force is applied. Therefore, additional research is necessary to determine the PDL stress range that should guide the treatment of impacted teeth with dilaceration.

The process of inverted ICI traction can increase the risk of palatal alveolar bone loss and result in root resorption [11, 15]. This study found a slight variation in the stress exerted on the alveolar bone surrounding the root due to various loading directions. In most models, the highest Von Mises stress in the root alveolar bone typically appears in the cervical region of the tooth. It is important to note that an IA of 20° does not occur at the cervical region but rather at the root apex. The analysis could potentially be associated with irregularities in the shape of tissues that support teeth or variations in the structure of different periodontal tissues.

The application of traction force plays a crucial role in determining the extent of apical displacement in orthodontic tooth movement [29, 30], which aligns with the findings of this study. In our investigation, we observed that as the traction angle decreases, there is a corresponding increase in apical displacement (vertical to the occlusal plane). Vardimon et al. [31]. thought that maintaining a 1:2 ratio between the rate of alveolar bone remodeling and the rate of tooth movement effectively reduces the risk of bone fenestration and dehiscence. Therefore, when dealing with initially inverted ICI, the effect of a continuous force on the PDL cannot be ignored. To quickly return the inverted ICI to its proper position, it is necessary to displace it perpendicular to the occlusal plane. It is recommended to apply gentle and intermittent force in the appropriate direction when dealing with an inverted ICI to minimize the risk of root resorption and bone damage, according to the IA.

Traditional orthodontic traction has shown favorable outcomes for inverted ICIs, but the fabrication process is cumbersome, and directional control is limited [10, 12, 32, 33]. In comparison, personalized digital design offers greater precision and shorter treatment times [34]. The digital traction appliance can preset the traction hook position for personalized traction, although its clinical application is still relatively limited. By integrating the results of FEA and digital design with CBCT, it is possible to ensure the accurate placement of orthodontic appliances and achieve effective correction of inverted ICI while ensuring patient safety. The integration of numerical design techniques such as digital traction appliances into orthodontic treatment offers significant advantages in treatment planning and execution. It improves overall treatment outcomes by providing the clinician with a comprehensive understanding of the forces involved and enables them to make informed decisions regarding the movement and positioning of the inverted ICI.

This study was limited to focusing solely on the momentary mechanical interactions that occur during the displacement of an impacted tooth and does not examine potential variations in these effects over time. It is worth mentioning that root resorption during actual treatments involves multiple biological processes, and advanced simulation methods are necessary to gain a deeper understanding of root resorption and alveolar bone remodeling within the context of tooth displacement. In the future, we will combine the research results with the digital design and play the importance of digital visualization of impacted teeth traction.

## Conclusions

With the limitations of this study, we can draw the following conclusions:

1. When dealing with larger inversion angles (more than 20°) of the inverted impacted central incisor, it is recommended to establish a traction direction at an approximate angle of 100°-110° to the anterior-superior angle of the crown.
2. For inversion angles smaller than 20°, it is possible to set a traction direction that is at a larger angle (110°-120°) to the crown length axis.

## Data Availability

No datasets were generated or analysed during the current study.

## Abbreviations

ICI: Impacted the central incisor
IA: Inverted angle
FD: Force direction
PDL: Periodontal ligament
CBCT: Cone-beam computed tomographic
3D: 3-dimensional
FEM: Finite element model
FEA: Finite element analysis
